# Frontiers in Preparations and Promising Applications of Mesoporous Polydopamine for Cancer Diagnosis and Treatment

**DOI:** 10.3390/pharmaceutics15010015

**Published:** 2022-12-21

**Authors:** Hualin Ma, Jingwen Peng, Jianing Zhang, Li Pan, Jiayi Ouyang, Zimu Li, Baochun Guo, Zhen Wang, Ying Xu, Daizheng Lian, Xiaowei Zeng

**Affiliations:** 1 Department of Nephrology, Shenzhen People’s Hospital (The Second Clinical Medical College, Jinan University, The First Affiliated Hospital, Southern University of Science and Technology), Shenzhen 518020, China; 2 School of Pharmaceutical Sciences (Shenzhen), Sun Yat-sen University, Shenzhen 518107, China; 3 Department of Hematology, Shenzhen People’s Hospital (The Second Clinical Medical College, Jinan University, The First Affiliated Hospital, Southern University of Science and Technology), Shenzhen 518020, China; 4 Department of Radiation Oncology, Shenzhen People’s Hospital (The Second Clinical Medical College, Jinan University, The First Affiliated Hospital, Southern University of Science and Technology), Shenzhen 518020, China

**Keywords:** nanomedicine, mesoporous polydopamine, polymerization mechanism, cancer diagnosis, cancer treatment

## Abstract

Polydopamine (PDA) is a natural melanin derived from marine mussels that has good biocompatibility, biodegradability, and photothermal conversion ability. As a new coating material, it offers a novel way to modify the surface of various substances. The drug loading capacity and encapsulation efficiency of PDA are greatly improved via the use of mesoporous materials. The abundant pore canals on mesoporous polydopamine (MPDA) exhibit a uniquely large surface area, which provides a structural basis for drug delivery. In this review, we systematically summarized the characteristics and manufacturing process of MPDA, introduced its application in the diagnosis and treatment of cancer, and discussed the existing problems in its development and clinical application. This comprehensive review will facilitate further research on MPDA in the fields of medicine including cancer therapy, materials science, and biology.

## 1. Introduction

Cancer is a substantial threat to human health, safety, and life. The rapid growth in incidence rate and mortality from cancer has brought immense challenges to modern medicine. The current therapeutic approaches to cancer care of surgery, chemotherapy, radiotherapy, and immunotherapy can achieve some results. However, these modalities have significant shortfalls including limitations in effectiveness as well as significant adverse effects. For example, with radiotherapy alone, the anti-radiation property of tumor tissue will lead to a decrease in efficacy, and even serious side effects [1]. During chemotherapy, cytotoxic drugs may directly kill tumor cells, but also affect normal cells. Traditional cancer treatment methods may produce beneficial effects in the initial stages, but drug resistance and cancer recurrence often occur in the longer term. Immunotherapy has substantially improved cancer treatment, but the complexity of the tumor microenvironment and specificity of the patient’s immune system mean that its efficacy remains suboptimal.

Therapeutic nanoparticles provide a new direction for cancer treatment [2,3,4]. By accurately targeting nanodrugs to the active site of tumor cells, unique drug-loading capacity, targeting ability, pharmacokinetics, and fluid dynamics can be achieved [5,6,7]. The widely used biomaterial, polydopamine (PDA), has great potential in drug delivery systems. It can be primed into polymeric nanoparticles targeted to deliver drugs to tumor sites and kill tumor cells through photothermal effects. However, PDA-based traditional nanomedicines have not produced the beneficial results expected. Jiang et al. reviewed nanomedicines in the treatment of breast cancer and reported that some nanomedicines only improve drug-loading capacity and have limited curative effect [8]. As an emerging nanodrug delivery carrier, mesoporous polydopamine (MPDA) has received considerably more attention than PDA in disease diagnosis and treatment. For example, Yuan et al. proposed an integrated phototherapy nanoplatform consisting of L-arginine, indocyanine green (ICG), and MPDA in a model of abscess formation, which showed effective biofilm elimination with an efficiency of about 100% [9].

**Figure 1:**
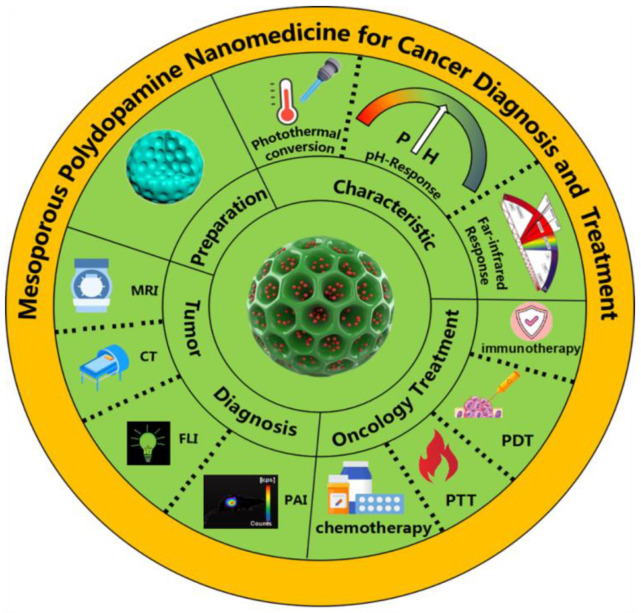
Schematic illustration of preparation, characterization, and tumor application of MPDA.

PDA has limited surface modification potential resulting in low drug loading potential and encapsulation inefficiency [10]. These problems can be mitigated by designing the porous structure of MPDA to form a multi-space coordination polymer layer with a more flexible structure. These results in improved efficacy and drug carrying capacity. As a nanodrug delivery system, MPDA amine has great potential in tumor diagnosis and treatment, which we have comprehensively and systematically summarized in this review. The preparation and polymerization mechanisms of different forms of MPDA are first introduced, then we describe the outstanding properties of MPDA. Finally, we explain in detail the application of MPDA in tumor management (Figure 1).

## 2. Polymerization Mechanisms of PDA

Three key methods of producing PDA are solution oxidation, enzymatic oxidation, and electropolymerization, with the solution oxidation method most widely used, due to its simple polymerization process [4,11].

**Figure 2:**
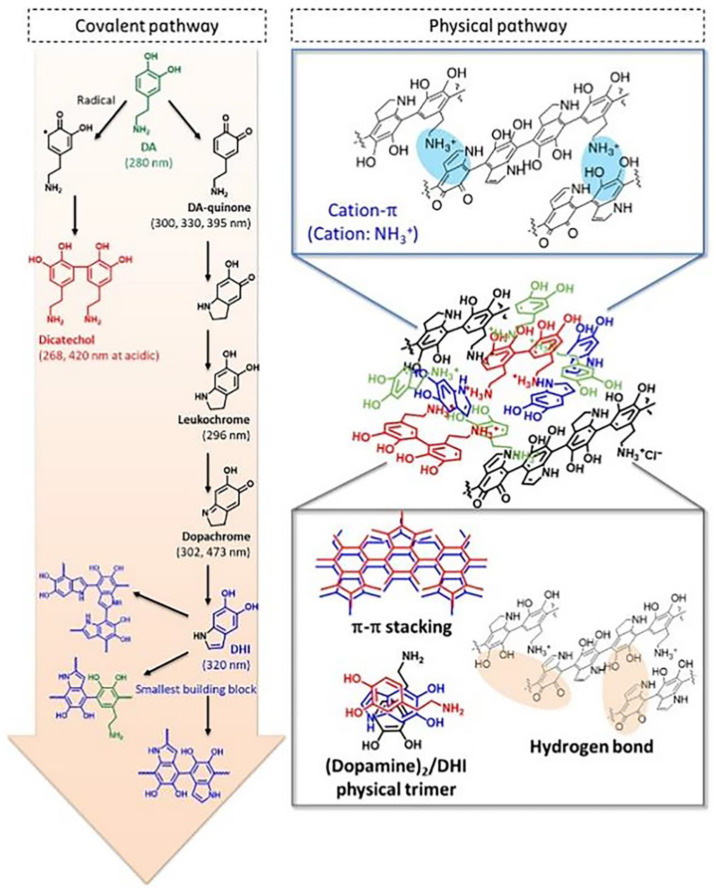
PDA synthesis pathways: covalent pathway and physical self-assembly pathway with main interaction. Reproduced with permission from ref. [13]. Copyright © 2018, American Association for the Advancement of Science.

Several theories have been proposed to explain the mechanisms underpinning how dopamine (DA) polymerizes into PDA. One of the most widely accepted models was suggested by Lee et al. and involves noncovalent self-assembly and covalent polymerization leading to the progressive assembly of PDA [11,12]. At the point of covalent bonding polymerization and under both aerobic and alkaline conditions, DA quinones are formed by oxidation of DA. After intramolecular cyclization and reversible oxidation, DA quinone is transformed into DA chrome and then rearranges intramolecularly into 5,6-dihydroxyindole (DHI). Abundant DA can be associated with DHI through non-covalent and produce self-assembled complexes including (DA)_2_/DHI. This process may be mainly attributed to intermolecular interactions including hydrogen bonding and π–π. Furthermore, the covalent oxidative products generated at the same time tightly wrap the self-assembled complex to finally form PDA. In further research, Lee and coworkers found that cation-π plays an important role in this progressive assembly as shown in Figure 2 [11,13,14].

## 3. Mesoporous Polydopamine Preparation

The templating method for synthesizing mesoporous carbon enables precise control of its properties including size, shape, and structure, but also has a simple preparation process to facilitate industrial production. The templating method includes hard and soft templating and contains three steps: preparation, template-directed synthesis, and removal. Material selection and removal are the key processes of hard templating; self-assembly and carbonization are essential for soft templating. The following content is an introduction to both templating methods [15].

### 3.1. Hard Templating

Rigid templates including high-molecular-weight polymers, molecular sieves, porous silicon, natural high-molecular-weight materials, carbon nanotubes, and colloidal crystals are most commonly used in hard templating. The hard templating synthesis strategy usually involves four steps: (i) preparation of a mesopore matrix with controlled architecture as a template; (ii) introduction of a carbon precursor into the mesopore matrix; (iii) polymerization of the carbon precursor and the mesopore matrix, and high-temperature carbonization; and (iv) removal of the template [16]. In this process, the crucial step for manageable synthesis is the filling of carbon precursors, which are influenced by carbon components’ random distribution, pore-clogging, and phase separation. Due to the rigidity and stability of the template, the mesoporous structure of the product is a counterpart of the mesopore matrix. Therefore, the mesopore matrix must be considered if aiming to control the product’s structure. Compared with soft templating, hard templating is time-consuming and complex because the mesopore matrix needs to be prepared and removed in the final synthesis stage. Additionally, the removal stage generally uses corrosive solvents, adding dangers to the synthesis process. As a template, MgO has structural, compositional, and thermal stabilities which means that it does not react with carbon precursors before carbonization temperature. Additionally, MgO can be removed by diluted acids such as acetic and citric acids, and it is nearly 100% recyclable [17]. The hard templating method’s outstanding features are its structural stability and controllability. The risk of using corrosive solvents can be mitigated by choosing a suitable template, but it remains unclear how to simplify the synthesis process and reduce the time requirements involved. 

### 3.2. Soft Templating

**Figure 3:**
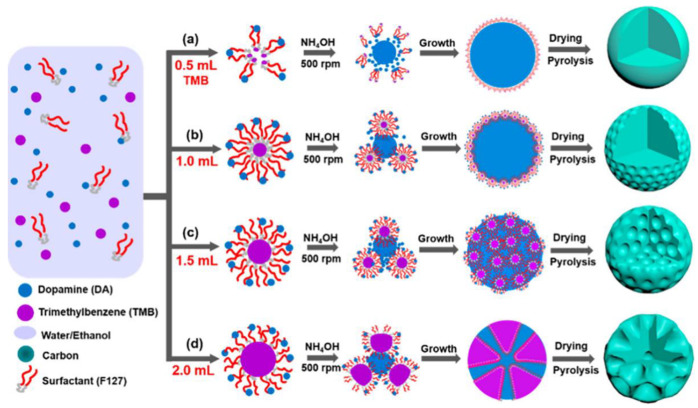
The schematic illustration of the formation process using the versatile nanoemulsion assembly approach to produce the N-doped mesoporous carbon nanospheres with various morphologies and mesostructures. Reproduced with permission from ref. [19]. Copyright © 2019, American Chemical Society.

Relatively soft surfactants such as high-molecular-weight block copolymers or molecular polymers are often used as soft templates. For example, triblock copolymers F127 or P123, cetyltrimethylammonium bromide, or polyoxymethylene-polybutene deblock copolymer, which have strong electrostatic and hydrogen bonding interactions with precursors, can be used to produce diverse morphology through relatively simple synthesis processes [15]. The chemical reaction between the template and the carbon precursor, the ratio of reactants, and the reaction temperature are important factors for templating. Compared with the hard template method, soft templating simplifies the synthesis process and causes less environmental pollution, but carries the disadvantages of a low template agent yield and low utilization rate. In recent years, the soft template method has been widely used in the multi-step assembly of multilayer mesoporous particles. Peng et al. described the synthesis of multifunctional yolk-shell-structured magnetic mesoporous PDA/carbon microspheres (Fe_3_O_4_@Void@mPDA) [18]. This group used Pluronic F127 (PEO–PPO–PEO) as a soft template and 1,3,5-trimethyl benzene (TMB) as a modifier to wrap the magnetic particles with a silica layer and form a stable nanoemulsion. Furthermore, DA underwent spontaneous oxidation polymerization to form an ordered F127/TMB/PDA composite shell on Fe_3_O_4_@nSiO_2_ in alkaline conditions. The silica layer and F127 were removed through a hot alkaline treatment and acetone extraction, and the Fe_3_O_4_@Void@MPDA that was produced showed perpendicular mesopores (5.2 nm), a high surface area (303.3 m^2^ g^−1^), and was rich in functional groups. In addition to the photothermal effect of PDA itself, the iron-oxide core’s photothermal effect can also play an important role in the near-infrared (NIR) light-triggered photothermal therapy. Studies have shown that the particle produced has a good photothermal effect with the limit temperature increasing from 32.3 to 57.8 C, the power increasing from 0.25 to 1.25 W cm^−2^, and the PDA shell providing it with good biocompatibility. Thus, it is an excellent photothermal agent. Furthermore, after 808 nm laser irradiation, the Fe_3_O_4_@Void@MPDA composite was shown to kill 95% of osteosarcoma cells K7M2-WT and U_2_O_2_, which was a better result than using Fe_3_O_4_ or MPDA particles alone (about 60% and 60%, respectively) under the same conditions. Collectively, it has proven that Fe_3_O_4_@Void@MPDA is an ideal agent to be employed in tumor photothermal therapy. In a further example, Gao et al. developed double-shelled hollow Ag_2_S@PDA nanocomposites [15]. These researchers used cetyltrimethylammonium bromide (CTAB) as a soft template to coat the HAg_2_S nanospheres with a silicon layer. They then utilized F127 and TMB to generate a PDA shell and removed the silicon layer by etching SiO_2_ in weak alkaline conditions. In contrast to normal mesoporous PDA particles, the double-shelled structure could provide a drug-loading space. The technique of producing mesoporous PDA with soft templates has been widely deployed in studies of mesoporous particles, and research on the effect of reaction conditions on the structure of the product has played an important role in this process. Peng et al. investigated use of a versatile nanoemulsion assembly approach for producing functional mesoporous carbon nanospheres with controllable sizes and structures. Their research has provided a theoretical basis for the application of such technologies [19]. F127 was used as the soft template and DA as the carbon propulsion. The amount of TMB was shown to directly affect the product size and structure because TMB mediated the interfacial interaction between F127 and DA. As shown in Figure 3, as the amount of TMB increased, the morphology of the micelle changed and the product gradually altered from smooth nanospheres to golf-shaped, multi-cavity, and dendritic nanospheres of 5 to 37 nm in diameter. The dendritic nanospheres had ultra-large mesopores (~37 nm), a relatively small diameter (~128 nm), and high specific surface area (635 m^2^ g^−1^). Further, they had excellent electrocatalytic activity, methanol tolerance, and long-term stability for the redox reaction. 

### 3.3. Preparation Conditions and Differences in Products

At present, the primary method for preparing mesoporous PDA is via the utilization of triblock copolymer Pluronic F127 and TMB as soft templates for synthesis. The approximate synthesis processes are as follows [20]. First, Pluronic F127 and TMB is dissolved in a water and ethanol solvent, then fully mixed with introduced TRIS and DA hydrochloride. After stirring at room temperature for 24h, product particles are separated by centrifugation, and the template is removed by extraction. Finally, the product particles are suspended in ethanol for further use. In this procedure, the TRIS buffer solution provides the alkaline conditions (pH ≈ 8.5) necessary for DA polymerization. To control product morphology, the proportion of Pluronic F127 and TMB can be adjusted as can the use of DA or the solvent, the reaction temperature, and/or the stirring speed.

#### The Proportion of Pluronic F127 and TMB, and Usage of DA

In work from Chen et al. the proportion of Pluronic F127 and TMB was controlled (TMB/F127 weight ratios from 0 to 20), and the product morphology changed from solid polydopamine nanospheres (PDANSs) into hollow polydopamine nanospheres (H-PDANSs), and from hollow mesoporous polydopamine nanospheres (H-MPDANSs) to mesoporous polydopamine nanospheres (MPDANSs) [21]. When only one of TMB or F127 was used, the product was only PDANSs. However, as the ratios of TMB/F127 were raised, H-PDANSs occurred and increased. When the TMB/F127 ratio reached 0.6, the main product became H-MPDANSs because TMB attained the necessary level to enter the F127 hydrophobic chains (PPO) and formulate H-MPDANSs. As the TMB/F127 weight ratio further increased from 0.75 to 0.95, the main product formed was H-MPDANSs with a few MPDANSs. The cavity sizes of H-MPDANs also gradually increased as the ratio increased. The limit value of the TMB/F127 weight ratio was 20, which was the largest ratio at which the emulsion droplets formed by TMB and F127 could maintain their pattern to produce H-MPDANSs. Use of DA also affected the morphology of the product. As the concentration of DA increased, the thickness of the PDA shell also increased, while the cavity size decreased and the H-MPDANS size remained largely unchanged. When the DA concentration reached 20 mg mL^−1^, PDA entered the TMB filling in the cavity of H-MPDANSs with the result being that the main product became MPDANSs.

Research by Peng et al. has revealed the influence of the solvent, reaction temperature, and stirring speed [19]. The morphology of the product is influenced by the ratio of water/ethanol. Aggregated nonporous particles are produced if the ratio of water/ethanol is too high or too low; irregular mesoporous particles are observed if the ratio is 7/3 or 3/7; uniform particles with dendritic PDA nanospheres that have ultra large pores are produced if the ratio is 1/1. This phenomenon occurs because the solvent influences the interfacial interaction of F127/TMB/PDA nanoemulsions to change the morphology of the product.

When the reaction temperature is 30 °C, the product is dendritic PDA nanospheres. At 40 °C, the swelling effect of TMB weakens and the product becomes uniform mesostructured PDA nanospheres. At a higher reaction temperature, the PDA nanospheres aggregate. The rate of DA polymerization increases and the stability of the nanoemulsions decreases can facilitate the degree of aggregation with increasing temperature. The degree of irregularity increases and the number of pore channels decreases while the stirring rate is raised from 250 to 750 rpm. The product turns from dense mesoporous particles to dendritic nanospheres, and finally to irregular shaped mesoporous particles with sparse pore channels. 

## 4. Properties of MPDA 

MPDA has excellent physical and chemical properties including metal ion coupling, surface modifiability, strong drug-carrying capacity, photothermal conversion, universal adhesion, biocompatibility, biodegradability, and responsiveness (e.g., pH response, far-infrared response). Inspired by natural melanin, PDA nanomaterials have a structure that promotes system surface activity. The presence of catechol groups that offer a high affinity for metal/metal oxides/metal sulfides with PDA led to the development of a new nanomaterial called MPDA [22]. Compared with PDA, the rich mesopores and cavities on the metal PDA nanomaterials have a uniquely large surface area, which provides a structural basis for drug transfer. In addition, the noncovalent coordination bond between PDA and metal ions can be destroyed by the photothermal conversion of MPDA. MPDA has excellent responsiveness in both near-infrared and tumor acidic conditions. Wang et al. endowed PDA with a porous structure that, in conjunction with tumor photothermal therapy (PTT), not only improved the near-infrared penetration ability but also realized the visualization function of PTT [23]. MPDA has outstanding biodegradability and biocompatibility due to its structural characteristics.

MPDA also has strong modifiability and improved performance with the introduction of other functional groups on the surface material. Lu et al. embedded the toll-like receptor (TLR) agonist imiquimod into MPDA to produce the dual characteristics of MPDA photothermal transformation and lymphatic-focused immune activation, playing an important role in anticancer immunotherapy [24]. Ding et al. established a reactive oxygen species (ROS) generator and used MPDA as a porous redox medium to exert long-term tumor inhibition under conditions of weak acidity and low hydrogen peroxide [25]. The powerful modifiability of MPDA can further improve performance and efficacy beyond that provided by PDA.

A variety of therapeutic methods and diagnostic functions can be combined by constructing a nano-drug delivery system for MPDA. Wu et al. reported that the multi-functional protein delivery system constructed by MPDA, with built-in plasma as nanoparticles, can release the functional protein in response to near-infrared light, with application in different biomedical fields [26]. Wang et al. constructed a multi-functional nanoplatform by embedding tungsten disulfide quantum dots (WS_2_QDs) into mesoporous DA nanosponges integrated with manganese dioxide, which can be used for thermal radiation therapy and tumor treatment detection [23].

In the widely accepted current model, levodopa undergoes noncovalent self-assembly and covalent polymerization, while PDA, a biomimetic polymer, is self-assembled under alkaline conditions. Due to the lack of templates, the surface area of such nanoparticles is very limited and they separate easily in the complex tumor microenvironment. Through further innovation, PDA can be used for modified mesoporous silica, which provides a stable framework and larger surface area to the structure as well as improving its physical and chemical properties. However, the transfer efficiency of such biomacromolecules is low due to the limitation of the actual size of mesoporous silicon [27]. Therefore, researchers have used high-molecular-weight block copolymer as a template to prepare mesoporous carbon materials, and then successfully synthesized MPDA. Compared with PDA, the pore diameters of MPDA are 2~50 nm, which improves both drug-loading capacity and the drug-releasing capacity of the nanocarriers [28]. 

PDA is the analogue of melanin that occurs naturally in living organisms. It can also be polymerized by DA, which is widely distributed in the human body and other biological systems. It has excellent biocompatibility and biodegradability, such that it essentially does not induce long-term toxicity in vivo. This is of great significance to the biomedical field. After being metabolized by enzymes such as monoamine oxidase, catecholamine methyltransferase, and aldehyde dehydrogenase in vivo, most of PDA is excreted by the kidneys into urine with low toxicity. Zhang et al. developed a paclitaxel (PTX)-loaded MPDA-polyethylene glycol (PEG) nanoplatform for combined photothermal therapy and chemotherapy [29]. The results showed that the nanoparticles eliminated the tumor completely in experimental nude mice. Further, routine blood biochemical tests showed no significant increase in liver and kidney indices at 48 h after injection, indicating that the nanoparticles did not cause any acute liver or kidney damage. Chen et al. designed a self-encapsulated nanoparticle with a mesoporous PDA core and a PDA shell for docetaxel loading that was modified with hyaluronic acid [30]. PDA is pH sensitive, and thus can be used to prevent drug release at off-target sites to enable targeted drug delivery. Both the chemotherapy system (M-D@P-H) and the PTT system (M@P-H+NIR) have excellent tumor growth inhibition, while combining chemotherapy with the PTT system (M-D@P-H+NIR) is even more effective, leading to almost complete tumor elimination.

## 5. Application of MPDA in Cancer Diagnosis and Therapy

MPDA possesses both the structural characteristics of mesoporous silicon and the dispersion modification and functional properties of polymers, and can be polymerized from PDA with excellent optical and electrical properties. Hence, MPDA has broad application prospects and research potential in cancer diagnosis and nanomedicine, while is widely used in the surface modification and functionalization of various materials. In recent years, mesoporous polydopamine has been used in tumor treatment, as shown in Table 1. In this section, we focus on the role of MPDA in cancer diagnosis and therapy.

### 5.1. Bioimaging

Biological imaging is the main method used to diagnose cancer. Early biobehavioral analysis and high-precision localization of tumors improves the accuracy of tumor qualitative and therapeutic analysis [38]. Hence, high-precision imaging plays an increasingly significant clinical role in personalizing medicine, including optimizing treatment effectiveness and monitoring response. At present, the commonly used biological imaging methods in clinical practice include magnetic resonance imaging (MRI), fluorescence imaging (FLI), photoacoustic imaging (PAI), computed tomography (CT) imaging, and positron emission tomography (PET) imaging. Multi-mode imaging combining various technologies has been developed and has the advantages of improved accuracy and convenience [39].

#### 5.1.1. MRI

MRI is a major tumor diagnostic tool, as it is noninvasive and has superior soft tissue resolution and an unrestricted penetration depth [40]. Contrast agents can enhance the distinction between biological targets and surrounding tissue, making diagnosis more accurate [41,42]. Commercially available MRI contrast agents come in two common forms: longitudinal relaxation (T1) and transverse relaxation (T2) [43]. Gadolinium (Gd) chelate is an example of a T1 positive contrast agent with high tissue resolution, which can enhance the T1 image signal by accelerating the longitudinal relaxation rate [44]. Superparamagnetic iron oxide (SPIO) nanoparticles are an example of a T2 negative contrast agent that enhances the reliability of soft tissue detection by providing a darker signal in T2 images via promotion of the transverse relaxation rate. However, single-mode contrast media has many shortfalls, mainly reflected in the lack of ability to detect small tumors accurately. Further, the T1 contrast agent, Gd, has a short blood circulation time, rendering it difficult to obtain high-resolution images. It also has a potential risk of adverse reactions [45]. T2 contrast agents also have challenges in clinical application, attributable to their negative contrast effect and susceptible artifacts. To overcome these difficulties, complementary diagnostic images can be obtained through the use of T1/T2 dual-mode contrast media, which greatly reduces false errors [46]. Predictably, therapeutic agents with dual T1- and T2-weighted MRI capabilities are promising candidates in the field of cancer therapy. 

Other MRI-active materials can be combined with PDA to give the prepared polymers contrast agent properties. Previous studies have found significant contrast enhancement in T2-weighted MRI of PDA-coated SPIO (PDA@SPIO) nanoparticles (NPs) compared with individual SPIO NPs, due to the high aggregation of SPIO magnetic NP nuclei [31]. In addition, PDA can provide an anchor for various metal ions through its excellent coordinate ability, which can be converted into PDA NPs by coating the PDA surface with ferric oxide or by absorbing metal ions (Fe^3+^, Fe^2+^, Gd^3+^, and Mn^2+^) [47]. Chen et al. proposed a T1/T2 MRI-guided administration modelled by a PDA-based coordination complex (PDA@CP_3_-DOX). The T1 contrast agent was an iron-chelated PDA nanoparticle, and the T2 contrast agent was a coordination polymer prepared from benzene-1,3,5-tricarboxylic acid (H_3_BTC) and iron. A platform for synergistic chemotherapy/photothermal therapy was established [32].

#### 5.1.2. FLI

FLI is widely used in disease diagnosis and image-guided therapy [48]. Drawbacks of FLI, such as extremely low radiation quantum yield and efficient ultraviolet and visible energy dissipation properties, have meant that PDA-based imaging platforms are less integrated. Thus, a new mode of integrating fluorescent molecules (dyes) into PDAs for FLI and sensing has been established [49]. Fluorescence–resonance energy transfer and photoinduced electron transfer may adversely affect the fluorescence intensity of the attached dye molecules, with PDA proven to be a fluorescence quencher [50].

A variety of PDA-based fluorescent nanomaterials have already been applied in the field of biological imaging due to their excellent luminescence properties. Zhang et al. first reported a fluorescent PDA with good biocompatibility. They destroyed the original functional groups and surface structure of PDA, and then used hydrogen peroxide to oxidize PDA NPs to obtain fluorescent PDA [51]. Zou et al. described a PDA-based nanoplatform that used a surface-initiated atom transfer radical polymerization mechanism while coupled with fluorescent Eu(III) complexes to obtain excellent FLI performance [52]. Monitoring the cellular internalization of fluorescent PDA in 4T1 cells revealed that PDA accumulated in the cytoplasm and fluorescence intensity increased with incubation time, indicating continuous PDA internalization. In addition, the fluorescence intensity of the liver increased significantly 8 h after intravenous injection, but gradually decreased 12 h after intravenous injection, showing a significant time-dependent distribution. These results indicated that fluorescent PDA has outstanding imaging performance and good biocompatibility.

#### 5.1.3. PAI

Photodiagnosis and phototherapy are widely used in cancer management, as they lack radiation effects and have high selectivity [53]. PDA has a large oxygen requirement and low penetration of the 980 nm light commonly used for irradiation, creating major obstacles to clinical application [54]. NIR photoresponsive nanoparticles can convert absorbed NIR light (808 nm) energy into heat, which is an essential basis for photoacoustic (PA) image-guided tumor photothermal therapy [55].

A range of nanomaterials have been reported to be light-responsive nanoprobes including noble metal nanomaterials, polymers, and carbon nanomaterials, which play an important role in PA imaging and PTT [56]. Liu et al. synthesized PDA-coated AuNBPs (AuNBPs@PDA), and further constructed a doxorubicin loaded AuNBPs@PDA-DOX nanoprobe, which improved PA imaging and the treatment efficiency of PTT. Significant enhancement of the PA signal was observed 24 h post-injection, identifying the excellent PA imaging performance of AuNBPs@PDA-DOX in vivo [33].

#### 5.1.4. CT Imaging

X-ray CT imaging, as the most widely used imaging technology in clinical practice, has the advantages of low cost and fast scanning speed. However, it also has the substantial limitations of low sensitivity and poor soft-tissue imaging capability. Hence, CT/MRI/PAI and other multi-mode imaging methods have been gradually developed to improve the accuracy and comprehensiveness of tumor management. Ding et al. prepared bowl-shaped gold@polydopamine yolk-shell NPs (bowl-shaped Au@PDA YNPs) as contrast agents for computed tomography/photoacoustic imaging for dual-mode image-guided chemotherapy and photothermal therapy. CT and PAI data demonstrated that the bowl-shaped Au@PDAYNPs tumor localization was valid [34]. 

### 5.2. Cancer Therapy

PDA has recently become widely used as a sustained-release drug delivery system [57]. It helps achieve targeted drug delivery, which can significantly reduce the toxic and adverse effects of tumor drugs on healthy physiological tissues, via its ability to remain stable at a neutral pH while degrading and releasing target drugs in an acidic tumor microenvironment [22].

However, traditional PDA nanoparticles have limited capacity for drug loading, and the drug capsule dosage form easily dissociates under the complex physiological environment, leading to poor drug effectiveness. In contrast, porous PDA nanoparticles have good therapeutic effect and a greatly improved drug-loading capacity. Chen et al. created MPDA nanoparticles using TRIS as a catalyst for DA polymerization, F127 and TMB as organic templates, and ethanol as a cosolvent [20]. A high DOX (doxorubicin hydrochloride) payload (up to 2000 µg/mg) has been shown using this nanocrystal drug delivery device. Both D-tocopherol polyethylene glycol 1000 succinate (TPGS, an inhibitor of ATP-binding cassette transporter) and doxorubicin were taken up by nanoparticles through hydrophobic–hydrophobic interactions and π–π stacking. Strong antitumor effects were seen in McF-7/ADR cells after exposure to doxorubicin produced from TPGS-mediated reversal of multidrug resistance. Thus, MPDA has good capacity for drug loading and sustained drug delivery, offering important development potential in drug dosage formulations [30]. 

Cheng et al. created a doxorubicin-delivery system for folic acid (FA)-conjugated PDA, where TPGS was injected into the PDA layer of mesoporous silica nanoparticles (MSNs) @PDA to create a multidrug lung cancer chemotherapy delivery system (MSNs-DOX@PDA-TPGS) in which FA could specifically bind folate receptors. By limiting the activity of P-glycoprotein (P-gp), TPGS could prevent cancer cells from developing multidrug resistance due to this transporter protein, thus increasing the therapeutic effect [57]. Nucleic acid aptamer was used as the targeting ligand in Cheng and colleagues’ PDA-based nanostructure for targeted drug delivery. A novel concept and point of reference for drug delivery in cancer therapy is provided by the efficient utilization of PDA as a delivery system vehicle for targeted dosage forms and combination therapy [58]. 

PDA can be loaded using physical adsorption or chemical interaction with active components include medicinal compounds, dyes, peptides, and DNA. It can also be combined with several different metal ions and radioisotopes concurrently. This means it can be used for synergistic therapies [59].

#### 5.2.1. Photothermal Therapy and Photodynamic Therapy (PDT)

Due to its low infectivity, low complication rate, spatiotemporal light-control properties, and quick recovery time, photothermal therapy is used as an alternative and complementary treatment to conventional cancer therapy, which may have more adverse effects and drug resistance. Strong absorption in the NIR band, minimal toxicity, and high biocompatibility are characteristics of a perfect photothermal agent [60]. However, transformation of absorbed light energy into heat when exposed to a NIR laser can raise local temperatures, harming cell membranes and denaturing proteins around cancer cells [61]. PDA shares similarities with natural eumelanin in terms of structure, and also has a greater photothermal conversion efficiency (40%) compared with the extensively employed photothermal reagent, gold nanorods. Further, PDA has significant ability to absorb in the NIR range [62,63,64].

**Figure 4:**
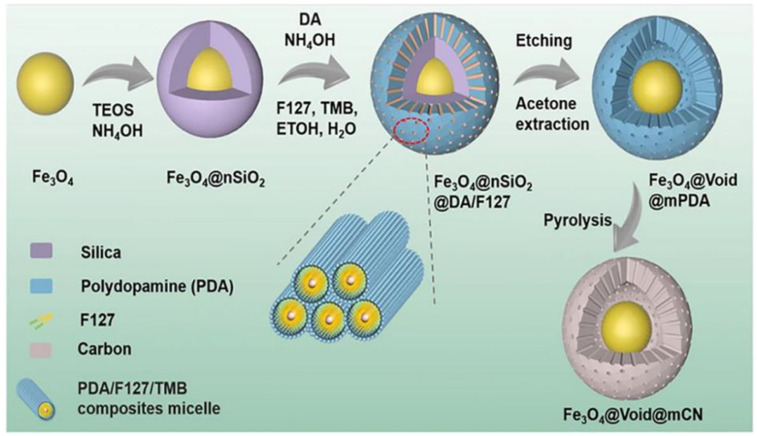
Preparation of Fe_3_O_4_, mPDA and derivative Fe_3_O_4_@mCN microspheres. Reproduced with permission from ref. [18]. Copyright © 2022, American Chemical Society.

PDA possesses exceptional adhesion, outstanding biocompatibility, substantial capacity for photothermal conversion, and an ability to scavenge reactive oxygen species. It is also rich in phenolic hydroxyl and amines. Strong magnetic resonance, the magnetothermal effect, and responsiveness are just a few of the unique chemical and physical characteristics of magnetic materials. Peng et al. [35]. integrated the PDA mesoporous structure with magnetic materials to create a system with a responsive intervoid magnetic core and a mesoporous yolk-shell. As shown in Figure 4 [35]. These magnetic mesoporous PDA (Fe_3_O_4_@Void@MPDA) microspheres and their carbon-based variants have a lot of interstitial space, high dispersion, and good biocompatibility. Ferric tetroxide and PDA are particularly good at absorbing light, while the yolk’s mesoporous structure helps dissipate heat. Fe_3_O_4_@Void@MPDA microspheres are an ideal photothermal agent because of their superior ability to suppress tumor cells. Based on this, additional synergistic therapy frequently enhances the therapeutic result.

PDT is another light-based method of drug therapy that has gained widespread recognition as an effective treatment for a variety of malignancies. This is because it is highly controllable, with few side effects including preserving organ function and reducing long-term morbidity. PDT requires three essential elements: light, oxygen, and the PDT agent (photosensitizer). The fundamental premise of this therapy is the generation of ROS due to the PDT agent’s interaction with oxygen when exposed to light. To overcome tumor resistance to PDT due to hypoxia, Liu et al. [65]. created a self-supplied O_2_ PDT system, with hemoglobin able to act as an in situ oxygen donor within it. PDA’s polyphenol units can serve as antioxidants, thus preventing Hb from being destroyed while in circulation. However, Hb readily oxidizes during blood circulation, which results in the loss of its ability to supply oxygen. Hb and DA monomers can be co-incubated to create PDA-Hb complexes quickly. Additionally, PDA has a lot of aromatic rings, which makes it possible to stack a range of aromatic chemicals, including aromatic PDT agents. Testing in vivo has revealed that the hybrid system could totally eradicate tumors and had a high PDT efficiency. 

#### 5.2.2. Photothermal Therapy Combined with Photodynamic Therapy

MPDA is simply applied in photodynamic and photo–heat combination treatment. During photodynamic treatment, photosensitizer drugs were required to convert molecular oxygen into harmful singlet oxygen and ROS (reactive oxygen species) [35,66]. ICG (Indocyanine Green) molecules could be employed as these photosensitizers in addition to being efficient photothermal agents. The loading of PDA-coated UCNP (up-conversion nanoparticles) (UCNPS-PDA-ICG) via physical adsorption was reported by Lin’s group [67] with in vitro testing showing a higher ROS generation efficiency than free ICG. Additionally, photothermal/photodynamic combination therapy showed more effective tumor reduction in vivo than following photodynamic therapy alone compared with photothermal therapy alone.

#### 5.2.3. Photothermal Therapy Combined with Immunotherapy 

Recent studies have demonstrated the effectiveness of combining photothermal and immune therapy using nanoparticles. PDA has natural NIR absorption strength and a high intensity photothermal conversion capability that can directly kill tumor cells, release tumor antigens, and improve tumor immunogenicity [68]. A nanoplatform supported by mesoporous silica nanoparticles (MSNs) was created by Huang and colleagues. This method effectively created the MSNs-ABC@PDA-OVA nanovaccine for combined photothermal/immunotherapy of melanoma by integrating the photothermal reagent PDA, the model antigen ovalbumin (OVA), and the antigen promoter ammonium bicarbonate (ABC). A strong immune memory was created, tumor recurrence and lung metastasis were prevented, and melanoma was totally eradicated with a cure rate of 75% after a single injection of MSNs-ABC@PDA-OVA [69]. 

A “two-in-one” synergistic tumor treatment has been developed by Xiao et al. using a mesoporous PDA delivery nanoplatform (IR-780@MPDA) loaded with NIR dye. IR-780 is a multipurpose medication that may be used for dual PA imaging-guided/NIR fluorescence photodynamic therapy and PTT (photothermal therapy). The transportability, water solubility, potential toxicity, and biocompatibility of IR-780 were all dramatically increased after loading on MPDA. In addition, synergistically PDT/PTT-induced ICD (immunogenic cell death) might activate CTLs (Cytotoxic T lymphocyte cells) at the tumor location, potentially inducing an immunotherapy response. Further, IR-780@MPDA caused synergistically targeted phototherapy leading to tumor eradication in vivo.

#### 5.2.4. Photothermal Therapy Combined with Chemotherapy 

To improve efficacy, approaches to cancer treatment have increasingly evolved from single agents to combination therapy. Combined interactions from two or more individual agents in a single nanostructure can produce synergistic therapeutic benefits that are far more potent than monotherapy. Photothermal chemotherapy is one of the potential methods for bimodal cancer treatment [70,71].

Cheng et al. demonstrated that PDA-PEG nanoparticles had good stability and effective medication retention under various physiological circumstances [36]. Pegylated PDA (PDA-PEG) nanoparticles have excellent photothermal capability and biocompatibility, which allows the efficient binding and stacking of anticancer medications like camptothecin and DOX for a combination approach. High photothermal conversion (41%) and paclitaxel (PTX) drug-loading content (paclitaxel, DLC = 15%) were present in the MPDA-PEG produced by Zhang and colleagues. The paclitaxel release rate was enhanced by a rise in temperature. Chemotherapy plus photothermal therapy (1 W cm^−2^ laser irradiation for 10 min) demonstrated an antitumor rate of up to 93% compared with chemotherapy or PTT alone [38]. This treatment had no clear systemic toxicity and also avoided the need to give prolonged photothermal therapy, which can result in overexpression of heat shock proteins and subsequent tumor thermal resistance.

**Figure 5:**
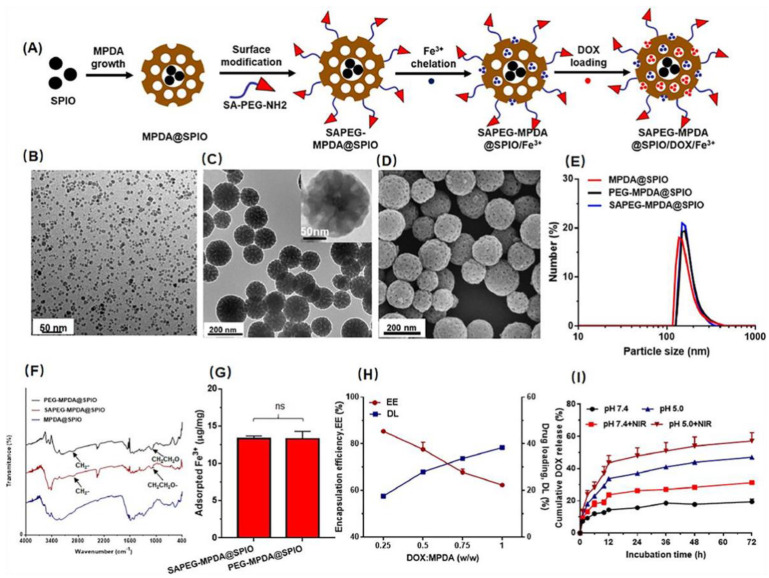
(**A**) Schematic illustration of the preparation of SAPEG-MPDA@SPIO/DOX/Fe^3+^ NPs. (**B**) TEM images of SPIO NPs. (**C**) TEM images of SAPEG-MPDA@SPIO NPs. (**D**) SEM images of SAPEG-MPDA@SPIO NPs. Particle size distribution (**E**) and FTIR spectra (**F**) of MPDA@SPIO, PEG-MPDA@SPIO and SAPEG-MPDA@SPIO NPs. (**G**) The amount of Fe^3+^ chelated by PEG-MPDA@SPIO NPs and SAPEG-MPDA@SPIO NPs (ns is non-significant, n = 3). (**H**) The DOX encapsulation efficiency (EE) and drug loading capability (DL) of SAPEG-MPDA@SPIO/Fe^3+^ NPs. (**I**) The DOX release profiles of SAPEG. MPDA@SPIO/DOX/Fe^3+^ NPs. Reproduced with permission from ref. [47]. Copyright © 2021, KeAi.

Shu et al. produced SAPEG-MPDA@SPIO/DOX/Fe^3+^ NPs as a novel T1/T2 bimodal MRI-guided chemotherapy–photothermal treatment for liver cancer. The findings from this research group demonstrated that SAPEG-MPDA@SPIO chelated with Fe^3+^ not only exhibited strong photothermal conversion ability and stability but also favorable T1-positive and T2-negative contrast enhancement [47]. By π–π stacking, DOX chemotherapy may be added to SAPEG-MPDA@SPIO/Fe^3+^ for pH/NIR dual response release behavior, as shown in Figure 5. Due to the combined chemotherapy and photothermal treatment, SAPEG-MPDA@SPIO/DOX/Fe^3+^ NPs can successfully limit tumor development when exposed to NIR radiation.

Finally, image-guided photothermal therapy and chemotherapy are also major applications of MPDA. Liu et al. showed that after two weeks of combination chemotherapy and photothermal therapy, the tumor volume was significantly reduced in vivo, courtesy of PDA NPs loaded with ICG, DOX, and Mn^2+^ ions [72]. 

#### 5.2.5. Chemotherapy Combined with Immunotherapy

Moreover, synergistic therapy can be achieved in cancer treatment by combining chemotherapy with MPDA and radiotherapy, such as 131I and 99mTc^3+^. For example, Liu’s group reported the development of up-conversion NPs (UCNP@PDA5-PEG) for PDA coatings [37]. A probe for multimodal imaging methods including magnetic resonance imaging, computed tomography, and up-conversion luminescence may be made using the UCNP core. These NPs can be chemically and photothermally treated to effectively prevent tumor growth and remove the tumor without it regenerating.

#### 5.2.6. Multi-Method Synergistic Treatment

Different forms of bimodal therapies have demonstrated superior anticancer efficacy compared with monotherapy. However, trimodal synergistic treatment, which is based on improved interactions of three separate therapies on a single nanostructure, can further increase therapeutic effectiveness. Conversion of endogenous hydrogen peroxide into a highly cytotoxic hydroxyl radical (•OH) can be facilitated using a metal Fenton catalyst in a process called chemodynamic therapy. This takes advantage of the overexpression of hydrogen peroxide by tumor cells relative to normal cells and the lack of need for oxygen. This somewhat selective susceptibility of tumor tissue to chemodynamic therapy spares healthy tissues from needless harm.

**Figure 6:**
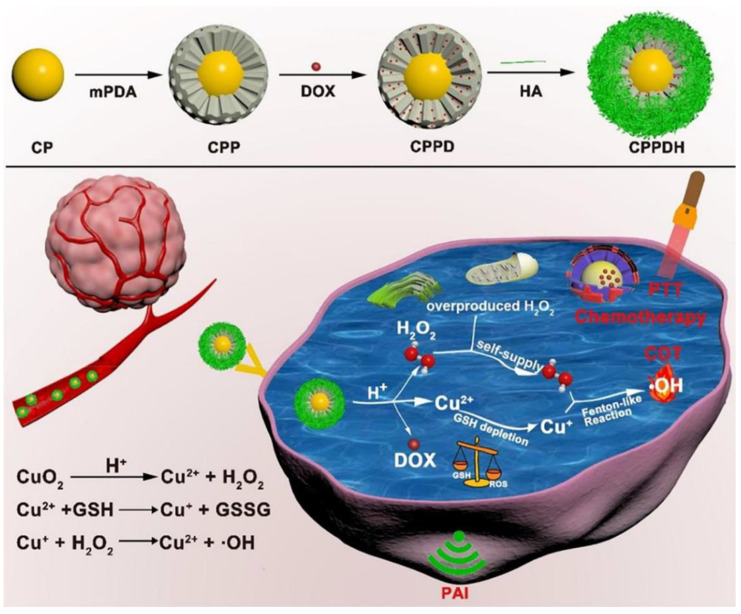
The synergistic auto-enhancing CDT, photothermal therapy, and chemotherapy that CPPDH uses as part of its therapeutic mechanism are shown in a schematic diagram. Reproduced with permission from ref. [35]. Copyright © 2021, American Chemical Society.

Xiao et al. described a self-contained hydrogen peroxide and GSH-consuming nanoplatform that combines the trio of chemodynamic, photothermal, and chemo-therapies. High drug-loading, a sizable photothermal impact, remarkable stability, and potent antitumor properties are all present in this nanoplatform [35]. The targeted HA allows the manufactured nanoplatform to efficiently concentrate at the tumor location after intravenous injection. Hydrogen peroxide self-sufficiency and Cu^2+^-induced GSH consumption both greatly increase the amount of •OH produced by the Cu+-driven Fenton-like reaction, as shown in Figure 6. Additionally, MPDA serves as a superb photoacoustic and photothermal reagent for photothermal treatment by laser irradiation and enables assessment of the targeting potential of nanoplatforms by PA imaging. In conjunction with photothermal treatment and chemotherapy, self-dilated •OH-mediated chemodynamic therapy exhibits good anticancer effects. No substantial systemic toxicity was noticed, offering a workable approach for tumor therapy.

**Figure 7:**
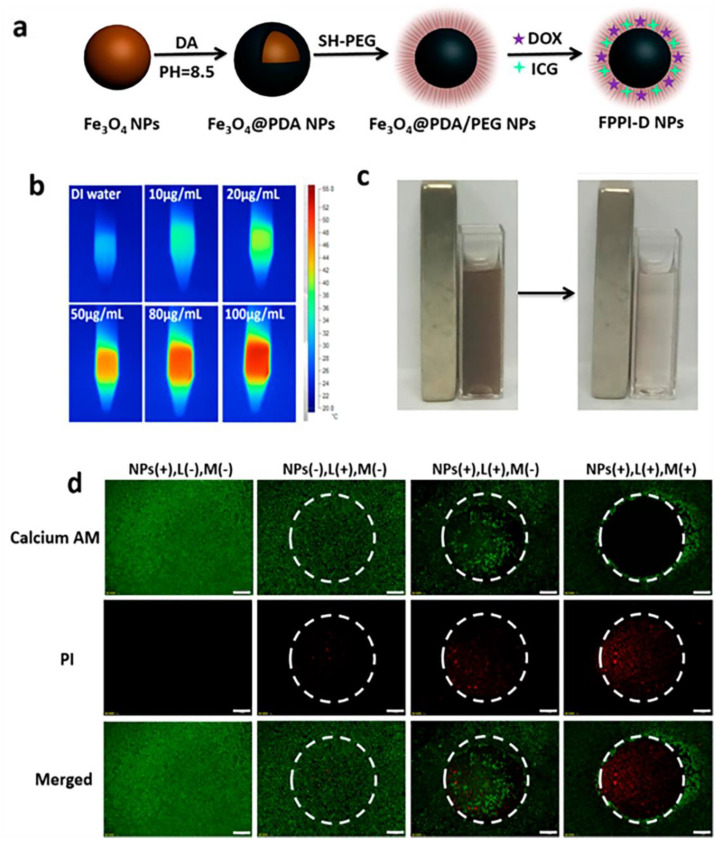
(**a**) Design of FPPI-DNPs. (**b**) Images from thermal imaging of test tubes containing samples of FPPI-DNPs at different concentrations. (**c**) FPPI-DNP aggregation in a magnetic field. (**d**) Live/dead cell staining following NPs, magnetic guiding (M), and NIR radiation therapy (L). The laser irradiation point is represented by the circular region in the image’s center. Scale bar: 500µm. Reproduced with permission from ref. [73]. Copyright © 2018, American Chemical Society.

Indocyanine green and doxorubicin-containing PDA-Fe_3_O_4_ core-shell NPs have been synthesized by Sun et al., as shown in Figure 7 [73]. HeLa cells are more likely to internalize when exposed to a strong superparamagnetic ferric tetroxide. ICG is a dye used in photothermal treatment that has significant NIR absorption. It also generates cytotoxic reactive oxygen species for photodynamic treatment at the same time. MPDA demonstrated a considerable tumor eradication result in the synergistic combination of magnetic field, photodynamic treatment, and drug therapy involving loading of the anti-cancer medication doxorubicin.

Guo et al. made a mesoporous TiO_2_-x-based nanoplatform (TiO_2_-x-DOX@PDA-Cy5.5) for the triad of photothermal therapy, photodynamic therapy, and chemotherapy [74]. Mesoporous TiO_2_-X nanoparticles make excellent NIR absorbers and can be employed in PTT/PDT combination applications as a photosensitizer. The mesoporous structure is suitable for drug loading during chemotherapy, the PDA layer permits NIR/pH-triggered drug release, and Cy5.5 may be used as a fluorescent imaging dye. Doxorubicin-induced DNA damage and the combined effect of PTT/PDT-induced membrane alterations and mitochondrial malfunction can significantly suppress tumor proliferation and totally eradicate malignancies.

## 6. Conclusions and Perspective

### 6.1. Significance of MPDA

MPDA has great potential and is slowly gathering momentum as having an important role in cancer therapy. This is due to its superior photothermal conversion properties compared with PDA, and its ability to incorporate high drug-loading properties and targeted drug delivery when combined with nanomaterials. Research into cancer treatments has always been of high interest, and the appearance of MPDA provides a novel idea to explore. This article has reviewed the synthesis, characteristics, and application of MPDA in the diagnosis and treatment of cancer. MPDA possesses both the structural characteristics of mesoporous materials and the functional properties of different polymers, in addition to having excellent optical and thermal properties. There is no doubt that MPDA, as a new drug delivery system, has become a hot topic in the field of cancer diagnosis and treatment and in nanomedicine. 

### 6.2. Challenges 

Although significant progress has been made in MPDA research in recent years, few such compounds have reached the clinic. Firstly, there should be more systematic studies on the structure, synthesis, biodegradability, and pharmacokinetics of MPDA materials. Meanwhile, its circulatory properties, such as in vivo clearance time, potential immunogenicity, and accumulation in tissues, must be elucidated before MPDA can be used in clinical practice. Furthermore, in order to new drugs to be used in healthcare, they must be shown to be effective and safe. While MPDA has excellent biocompatibility, there are also studies claiming that PDA may result in toxicity [75]. Currently, the biological application of MPDA is still in the cell and animal experimental stage, which lacks human testing. 

### 6.3. Perspective

As nanotechnology and tumor therapeutics continue to advance, the potential for clinical application of MPDA in cancer treatment is expected to improve. Based on the excellent photothermal conversion ability of PDA, combining photothermal therapy with other therapeutic methods will be the main and essential research interest of MPDA in clinic application. By preparing the MPDA delivery nanoplatform, we can add different kinds of anticancer drugs into the pore to achieve a wide range of applications. Meanwhile, tumor cell-related biomarkers can also be conjugated on the PDA films to trigger immunotherapy [76].

In summary, we ardently anticipate that this review will provide readers with basic and cutting-edge research information about MPDA, and ignite researchers’ passion to conduct further research on the synthetic design and clinical application of MPDA, which will promote the application of MPDA in tumor diagnosis, therapy, and other fields.

## Figures and Tables

**Table 1:** Mesoporous polydopamine applied in tumor treatment.

Formulation	Drug	Cancer	Imaging	Therapy	Ref.
Nanoparticles	Imiquimod	B16-F10		PTT–Immunotherapy	[24]
Nanoparticles	Paclitaxel	A549	PA	Chemo–photothermal therapy	[29]
Nanoparticles	Docetaxel	4T1		Chemo–photothermal therapy	[30]
Nanoparticles	Doxorubicin	HeLa	MRI	Chemo–photothermal therapy	[31]
Nanoparticles	Doxorubicin	HeLa	MRI	Chemo–photothermal therapy	[32]
Nanoparticles	Doxorubicin	4T1	PA	Chemo–photothermal therapy	[33]
Nanoparticles	Doxorubicin	HepG-2	PA/CT	Chemo–photothermal therapy	[34]
Nanoparticles	Doxorubicin	K7M2-WT, U2OS		Chemo–photothermal/Chemodynamic therapy	[35]
Nanoparticles	Doxorubicin	CT26	MRI	Chemo–photothermal therapy	[36]
Nanoparticles	Doxorubicin	HeLa	MRI	Chemo–photothermal therapy	[37]

## Data Availability

Not applicable.
